# Nutrient-Element-Mediated Alleviation of Cadmium Stress in Plants: Mechanistic Insights and Practical Implications

**DOI:** 10.3390/plants14193081

**Published:** 2025-10-06

**Authors:** Xichao Sun, Liwen Zhang, Yingchen Gu, Peng Wang, Haiwei Liu, Liwen Qiang, Qingqing Huang

**Affiliations:** 1 Agro-Environmental Protection Institute, Ministry of Agriculture and Rural Affairs, Tianjin 300191, China; sunxichao@caas.cn; 2 Key Laboratory of Tobacco Biology and Processing, Ministry of Agriculture and Rural Affairs, Tobacco Research Institute, Chinese Academy of Agricultural Sciences, Qingdao 266101, China; zlwwgg@163.com (L.Z.); gyczc201@163.com (Y.G.); wangpeng03@caas.cn (P.W.); heaveyliu@163.com (H.L.)

**Keywords:** cadmium toxicity, nutrient homeostasis, metal transporters, chelation, antioxidants, compartmentalization, signal transduction, gene regulation, phytoremediation, food safety

## Abstract

Cadmium (Cd), a pervasive and highly phytotoxic metal pollutant, poses severe threats to agricultural productivity, ecosystem stability, and human health through its entry into the food chain. Plants have evolved intricate defense mechanisms, among which the strategic manipulation of nutrient elements emerges as a critical physiological and biochemical strategy for mitigating Cd stress. This comprehensive review delves deeply into the multifaceted roles of essential macronutrient elements (nitrogen, phosphorus, potassium, calcium, magnesium, sulfur), essential micronutrient elements (zinc, iron, manganese, copper) and non-essential beneficial elements (silicon, selenium) in modulating plant responses to Cd toxicity. We meticulously dissect the physiological, biochemical, and molecular underpinnings of how these nutrients influence Cd bioavailability in the rhizosphere, Cd uptake and translocation pathways, sequestration and compartmentalization within plant tissues, and the activation of antioxidant defense systems. Nutrient elements exert their influence through diverse mechanisms: competing with Cd for root uptake transporters, promoting the synthesis of complexes that reduce Cd mobility, stabilizing cell walls and plasma membranes to restrict apoplastic flow and symplastic influx, modulating redox homeostasis by enhancing antioxidant enzyme activities and non-enzymatic antioxidant pools, regulating signal transduction pathways, and influencing gene expression profiles related to metal transport, chelation, and detoxification. The complex interactions between nutrients themselves further shape the plant’s capacity to withstand Cd stress. Recent advances elucidating nutrient-mediated epigenetic regulation, microRNA involvement, and the role of nutrient-sensing signaling hubs in Cd responses are critically evaluated. Furthermore, we synthesize the practical implications of nutrient management strategies, including optimized fertilization regimes, selection of nutrient-efficient genotypes, and utilization of nutrient-enriched amendments, for enhancing phytoremediation efficiency and developing low-Cd-accumulating crops, thereby contributing to safer food production and environmental restoration in Cd-contaminated soils. The intricate interplay between plant nutritional status and Cd stress resilience underscores the necessity for a holistic, nutrient-centric approach in managing Cd toxicity in agroecosystems.

## 1. Introduction

Cadmium (Cd) contamination of agricultural soils represents a global environmental challenge of escalating magnitude, primarily originating from anthropogenic activities such as industrial emissions (smelting, electroplating), mining operations, application of phosphate fertilizers and sewage sludge, and improper disposal of electronic waste [1,2]. The most significant Cd contamination in agricultural soils occurs in regions including northern and central India, Pakistan, Bangladesh, southern China, southern Thailand and Cambodia, Iran, Türkiye, Ethiopia, Nigeria, South Africa, Mexico, and Cuba [3]. Unlike essential micronutrients, Cd serves no known biological function in plants and is readily absorbed by roots through pathways designed for essential divalent cations like ferrous ion (Fe^2+^), zinc ion (Zn^2+^), calcium ion (Ca^2+^), and manganese ion (Mn^2+^) [4]. Once inside the plant, Cd disrupts a plethora of physiological processes. It induces severe oxidative stress by disrupting cellular redox homeostasis, which releases free Fe^2+^ that catalyzes reactive oxygen species (ROS) production via Fenton and Haber–Weiss reactions, and by inactivating antioxidant enzymes [5]. Cd impairs photosynthesis by damaging chloroplast structure, inhibiting chlorophyll biosynthesis, and interfering with photosynthetic electron transport and Calvin cycle enzymes [6]. It disrupts water and nutrient uptake by affecting root architecture and membrane integrity/permeability [7], interferes with nitrogen (N) metabolism and enzyme activities [8], and can cause DNA damage and trigger programmed cell death under severe stress [9]. Consequently, Cd accumulation in edible plant parts poses significant risks to human health, including renal dysfunction, bone demineralization (Itai-Itai disease), and increased cancer risk [10]. Plants vary drastically in Cd uptake capacity: hyperaccumulators including *Noccaea caerulescens* and *Sedum alfredii* accumulate extraordinary Cd levels in shoots, while staple crops such as rice (*Oryza sativa*) and wheat (*Triticum aestivum*) readily translocate Cd to edible grains. This accumulation not only reduces crop yield and quality but also propagates Cd through food webs, threatening ecosystem stability and human health via chronic exposure [11]. Mitigating Cd uptake and toxicity in plants is therefore paramount for ensuring food safety and ecosystem health. Plants deploy a hierarchical defense strategy against Cd, encompassing exclusion (limiting root uptake), sequestration (binding Cd in roots or vacuoles), and tolerance (detoxification via chelation and antioxidant systems) [12,13]. Crucially, the status and availability of essential plant nutrients profoundly influence each tier of this defense strategy. Nutrient elements can modulate Cd behavior in the soil-plant continuum, interact with Cd at cellular and subcellular levels, and regulate the expression and activity of genes and proteins involved in Cd detoxification. This review comprehensively examines the intricate mechanisms by which nutrient elements govern plant responses to Cd stress, synthesizing recent advances in physiological, biochemical, and molecular understanding, and evaluating the translational potential of nutrient management for sustainable Cd mitigation.

## 2. Cd in the Soil–Plant System: Bioavailability and Toxicity Pathways

The phytoavailability of Cd is governed by complex interactions between soil properties (pH, redox potential, organic matter content, clay mineralogy, cation exchange capacity) and rhizosphere processes [14,15]. Cd exists primarily as cadmium ion (Cd^2+^) in soil solution under aerobic conditions. Soil acidification (low pH) significantly increases Cd solubility and bioavailability, while liming (increasing pH) promotes its adsorption onto soil colloids (clays, iron/manganese (Fe/Mn) oxides, organic matter) and precipitation as carbonates or phosphates, reducing phytoavailability [16,17]. Soil organic matter (SOM) can both increase Cd mobility through the formation of soluble organic complexes or decrease it via adsorption onto solid organic phases [18]. Root exudates, including low-molecular-weight organic acids (e.g., citrate, malate, oxalate), phytosiderophores (under Fe deficiency), and protons, dramatically alter rhizosphere chemistry, influencing Cd speciation and solubility [19]. For instance, citrate can form soluble complexes with Cd, potentially enhancing mobility, while phosphate exudation might promote Cd precipitation [20]. Microbially mediated transformations in the rhizosphere also play critical roles [21].

**Figure 1:**
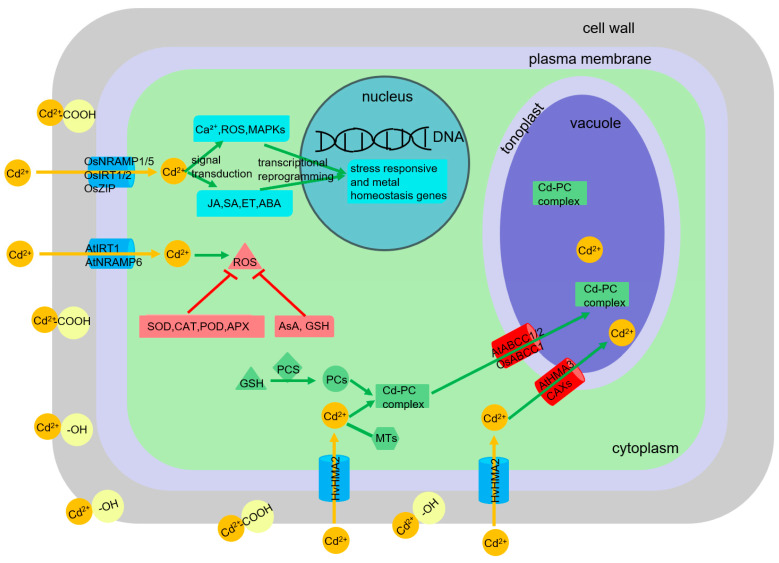
Mechanism diagram of Cd detoxification in plant root cells.

Upon entering the root symplast, primarily via transporters for essential cations (e.g., OsNramp1/5, OsIRT1/2, OsZIP transporters in rice; AtIRT1, AtNramp6 in *Arabidopsis*; HvHMA2 in barley), Cd faces the plant’s internal detoxification machinery [22,23,24]. Key processes include: (1) Chelation by thiol-rich peptides: Phytochelatins (PCs), enzymatically synthesized from glutathione (GSH) by phytochelatin synthase (PCS), form high-affinity Cd-PC complexes, which are then sequestered into vacuoles by tonoplast ABC transporters (e.g., AtABCC1/2, OsABCC1) [25]. Metallothioneins (MTs), gene-encoded cysteine-rich proteins, also contribute to Cd binding, particularly in certain plant families such as Brassicaceae (crucifers) and Fabaceae (legumes) [26,27]. (2) Compartmentalization: Vacuolar sequestration is a primary mechanism for isolating Cd away from sensitive cytoplasmic sites. Tonoplast-localized transporters like HMAs (e.g., AtHMA3, which transports free Cd^2+^ into vacuoles) and Cation/H^+^ exchangers (CAXs) facilitate this process [28]. (3) Antioxidant defense: Cd-induced ROS necessitate a robust antioxidant response involving enzymatic scavengers including superoxide dismutase (SOD), catalase (CAT), peroxidase (POD), ascorbate peroxidase (APX), glutathione reductase (GR) and non-enzymatic antioxidants including ascorbic acid (AsA), GSH, phenolics, carotenoids, tocopherols [29]. The AsA-GSH cycle is central to H_2_O_2_ detoxification. (4) Cell wall binding: The apoplast, particularly the cell wall rich in pectin, hemicellulose, and lignin, can act as a significant sink for Cd ions through cation exchange and complexation with carboxyl and hydroxyl groups [30]. (5) Hormonal signaling and gene regulation: Cd stress triggers complex signaling cascades involving Ca^2+^, ROS, mitogen-activated protein kinases (MAPKs), and phytohormones including jasmonic acid (JA), salicylic acid (SA), ethylene (ET), abscisic acid (ABA), leading to transcriptional reprogramming of stress-responsive and metal homeostasis genes [31,32] (Figure 1). The perturbation of essential nutrient homeostasis by Cd is a critical facet of its toxicity, disrupting vital metabolic functions. Conversely, optimizing nutrient supply represents a potent lever to counteract Cd toxicity by interfering with these uptake and toxicity pathways at multiple levels.

## 3. Regulation of Cd Stress by Essential Macronutrient Elements

### 3.1. Nitrogen

N exerts profound and complex influences on Cd accumulation and tolerance, mediated through its forms (ammonium (NH_4_^+^) vs. nitrate (NO_3_^−^)), availability, and profound impact on plant growth, metabolism, and rhizosphere chemistry [33]. NO_3_^−^ fertilization often leads to rhizosphere alkalinization due to preferential uptake with H^+^ or association with OH^−^/HCO_3_^−^ efflux. This pH increase generally reduces Cd solubility and bioavailability in the soil [34]. Conversely, NH_4_^+^ nutrition typically causes rhizosphere acidification (H^+^ efflux during NH_4_^+^ assimilation), potentially increasing Cd mobility and uptake [35]. Comparative studies frequently report lower Cd accumulation in plants supplied with NO_3_^−^ compared to NH_4_^+^ under Cd stress [36]. Beyond pH effects, N form directly influences transporter expression. NH_4_^+^ uptake via ammonium transporters (AMTs) may indirectly affect Cd^2+^ influx, while NO_3_^−^ uptake via nitrate transporters (NRTs) can influence Cd distribution [37,38]. Crucially, N is a fundamental constituent of key Cd-detoxifying molecules. Glutamine synthetase/glutamate synthase (GS/GOGAT) cycle activity, central to N assimilation, produces glutamate, the precursor for GSH synthesis [39]. Adequate N supply enhances GSH and PC production, thereby improving Cd chelation and sequestration [40]. For example, N application increased root PC synthesis and vacuolar sequestration of Cd in *Solanum nigrum*, enhancing its Cd tolerance and accumulation capacity for phytoremediation [41]. Furthermore, N is integral to numerous enzymes involved in antioxidant defense and photosynthesis. Optimal N status maintains photosynthetic efficiency and carbon supply for defense compound synthesis while bolstering the enzymatic antioxidant machinery against Cd-induced oxidative damage [42]. Recent transcriptomic and proteomic analyses reveal that N availability modulates the expression of genes encoding metal transporters (e.g., Nramps, HMAs), PCS, antioxidant enzymes (SOD, APX, GR), and stress-responsive transcription factors under Cd exposure [43,44]. The intricate interplay between N metabolism, particularly GSH synthesis, and Cd detoxification highlights N management as a critical factor in Cd-stressed plants.

### 3.2. Phosphorus

Phosphorus (P) exhibits a dualistic relationship with Cd in plants, involving both soil chemistry interactions and physiological antagonism within the plant. In the soil, phosphate application can significantly reduce Cd bioavailability through the precipitation of highly insoluble Cd phosphate minerals (e.g., Cd_5_(PO_4_)_3_OH, Cd_3_(PO_4_)_2_) [45,46]. This is particularly effective in neutral to alkaline soils. Furthermore, P fertilization can increase soil adsorption capacity and stimulate microbial activity that may immobilize Cd [47]. Consequently, P application often decreases Cd concentration in plant tissues, especially roots and shoots, by limiting soil-root transfer [48]. However, this effect is soil-dependent and can be less pronounced or even reversed in acidic soils where phosphate solubility is low [49]. Within the plant, P and Cd exhibit physiological antagonism. Cd can disrupt P uptake and metabolism by interfering with phosphate transporters (e.g., PHT1 family) and inhibiting key P-metabolizing enzymes like acid phosphatases [50]. Conversely, adequate P nutrition can ameliorate Cd toxicity. P is a vital component of energy metabolism (ATP), nucleic acids, phospholipids (membrane integrity), and coenzymes. Sufficient P supply helps maintain cellular energy status crucial for Cd detoxification processes (e.g., ATP-dependent transporters like HMAs and ABCCs for vacuolar sequestration), stabilizes membranes compromised by Cd-induced lipid peroxidation, and supports nucleic acid stability [46,51]. Enhanced P status can also stimulate root growth, potentially increasing the root surface area for nutrient uptake while diluting Cd concentration [52]. Studies demonstrate that P application enhances antioxidant defense (increased activities of SOD, POD, CAT) and improves photosynthetic performance in Cd-stressed plants [53]. Molecular studies indicate that P deficiency can upregulate certain PHT transporters with potential affinity for Cd or Cd-P complexes, suggesting complex regulation at the transporter level [54]. Optimizing P management, considering soil properties and plant demand, is thus essential for minimizing Cd uptake and toxicity.

### 3.3. Potassium

Potassium (K), the primary osmoticum and cation regulator in plants, plays a significant role in mitigating Cd stress through its involvement in stomatal regulation, enzyme activation, charge balance, and membrane potential maintenance. Cd stress often induces potassium ion (K^+^) leakage from roots and shoots due to membrane damage and disruption of K^+^ channels and transporters (e.g., AKT1, SKOR) [55,56]. This K^+^ efflux exacerbates cellular ionic imbalance and osmotic stress. Adequate K^+^ supply counteracts this by maintaining membrane integrity and H^+^-ATPase activity, reducing Cd-induced K^+^ efflux and improving plant water status [57]. K^+^ is a crucial cofactor for over 50 enzymes, including key players in carbohydrate metabolism, protein synthesis, and, importantly, ROS-scavenging enzymes like SOD [58]. Enhanced K^+^ nutrition can therefore bolster the antioxidant capacity of plants under Cd stress, reducing ROS accumulation and oxidative damage markers like malondialdehyde (MDA) [59]. K^+^ also competes with Cd^2+^ for binding sites on the cell wall and plasma membrane due to their similar hydrated ionic radii, potentially reducing Cd adsorption and influx into the symplast [60]. Furthermore, K^+^ contributes to charge balance during the uptake of anions (like NO_3_^−^) and influences xylem loading. Adequate K^+^ promotes xylem sap flow and can influence the partitioning of Cd between roots and shoots, sometimes reducing shoot Cd concentration by enhancing root retention or reducing root-to-shoot translocation [61,62]. Transcriptomic analyses reveal that K^+^ application modulates the expression of genes involved in Cd transport (e.g., *HMA* genes for efflux/sequestration), antioxidant defense, and general stress responses [63]. K management, therefore, contributes significantly to maintaining cellular homeostasis and reducing Cd sensitivity.

### 3.4. Calcium

Calcium (Ca) serves as a ubiquitous secondary messenger and a vital structural component of cell walls and membranes. Its role in Cd stress mitigation is multifaceted and central [64]. Ca^2+^ competes directly with Cd^2+^ for binding sites on the root cell wall (pectin, exchange sites) and plasma membrane transporters (e.g., voltage-independent cation channels, annexins) due to their similar charge and hydrated radius [65]. Exogenous Ca application consistently reduces Cd uptake across numerous plant species by saturating these sites and reducing Cd influx [66]. Furthermore, Ca stabilizes plasma membrane structure and function by bridging phospholipids and proteins, counteracting Cd-induced membrane lipid peroxidation and permeability [67]. This preserves membrane integrity, reducing Cd influx and ion leakage. Crucially, Ca^2+^ is a key component in signal transduction cascades triggered by abiotic stresses, including Cd. Cd stress induces cytosolic Ca^2+^ ([Ca^2+^]ₘ) spikes originating from influx through plasma membrane channels (e.g., cyclic nucleotide-gated channels, glutamate receptor-like channels) and release from internal stores [68,69]. These [Ca^2+^]ₘ signatures are decoded by Ca^2+^ sensors, including calmodulin (CaM), calmodulin-like proteins (CMLs), calcium-dependent protein kinases (CDPKs), and calcineurin B-like proteins (CBLs) interacting with CBL-interacting protein kinases (CIPKs) [70,71]. This Ca^2+^ signaling network activates downstream responses, including the expression of genes encoding antioxidant enzymes (e.g., SOD, CAT, POD), metal transporters (e.g., HMAs, Nramps, MTPs), and PCS [72]. For instance, the CBL-CIPK pathway regulates the activity of transporters like MTP8 in response to toxic metal [73]. Adequate Ca supply enhances the plant’s ability to generate and interpret these stress signals effectively. Ca also promotes callose deposition at plasmodesmata, potentially limiting symplastic Cd movement [74]. Maintaining optimal Ca nutrition is therefore fundamental for both physical barrier function against Cd entry and the activation of complex signaling-based defense responses.

### 3.5. Magnesium

Magnesium (Mg), the central atom of the chlorophyll molecule and an essential activator for numerous enzymes (especially those utilizing ATP), plays a vital but sometimes overlooked role in Cd stress responses. Cd toxicity often induces Mg deficiency symptoms, as Cd can interfere with Mg uptake and translocation [75]. This competition occurs at root uptake sites and potentially within the plant, disrupting Mg-dependent physiological processes. Mg deficiency exacerbates Cd toxicity by impairing photosynthesis (reduced chlorophyll content and rubisco activity), weakening the antioxidant system, and destabilizing ribosome structure, thus inhibiting protein synthesis [76,77]. Conversely, adequate Mg supply alleviates Cd toxicity. Mg competes with Cd for uptake sites on roots and within tissues, reducing Cd accumulation, particularly in sensitive photosynthetic tissues [78]. Mg is crucial for maintaining the structural integrity of chloroplasts and thylakoid membranes. Sufficient Mg helps protect the photosynthetic apparatus from Cd-induced damage, sustaining carbon fixation and energy production necessary for Cd detoxification processes (e.g., GSH/PC synthesis, active transport) [79]. Mg also acts as a cofactor for enzymes involved in GSH synthesis (γ-glutamylcysteine synthetase, glutathione synthetase) and regeneration (glutathione reductase) [80]. Enhanced Mg status can therefore boost the cellular GSH pool, crucial for both direct Cd chelation and PC synthesis, as well as for the AsA-GSH cycle central to H_2_O_2_ detoxification. Studies show that Mg application reduces Cd-induced ROS accumulation (H_2_O_2_, O_2_^−^) and lipid peroxidation, while increasing the activities of SOD, CAT, APX, and GR, and the levels of AsA and GSH [81]. Mg also contributes to the stabilization of nucleic acids and membranes. Optimizing Mg nutrition is essential for maintaining core metabolic functions and enhancing the biochemical resilience of plants under Cd stress.

### 3.6. Sulfur

Sulfur (S) metabolism is inextricably linked to Cd detoxification, primarily through the biosynthesis of cysteine-rich compounds. S assimilation, initiated by ATP sulfurylase and adenosine 5‘-phosphosulfate (APS) reductase, produces cysteine, the fundamental building block for GSH and PCs [82]. GSH, a key cellular antioxidant and redox buffer, is synthesized via γ-glutamylcysteine synthetase (γ-ECS) and glutathione synthetase (GS) [83]. Crucially, under Cd exposure, GSH serves as the substrate for PCS, which catalyzes the polymerization of GSH into phytochelatins [84]. PCs exhibit an exceptionally high affinity for Cd^2+^, forming low-molecular-weight Cd-PC complexes. These complexes are then actively transported into the vacuole by ATP-binding cassette (ABC) transporters (e.g., AtABCC1/2, OsABCC1) for long-term storage, effectively detoxifying cytosolic Cd [85]. Consequently, S availability is a major determinant of PC production capacity and Cd tolerance. S deficiency severely compromises GSH and PC synthesis, rendering plants hypersensitive to Cd [86]. Conversely, S fertilization dramatically enhances Cd tolerance and accumulation (in hyperaccumulators) or exclusion by promoting the synthesis of thiol compounds for root sequestration [87]. S supply also enhances the synthesis of other S-containing defense compounds like methionine, S-adenosylmethionine (SAM), glucosinolates, and various sulfated compounds, which may contribute indirectly to Cd tolerance through antioxidant or chelating properties [88]. Furthermore, S nutrition influences the expression of genes encoding sulfate transporters (SULTRs), enzymes of S assimilation (adenosine triphosphate sulfurylase (ATPS), adenosine 5′-phosphosulfate reductase (APR)), GSH synthesis (GSH1, GSH2), and PC synthesis [89]. S status also impacts the cellular redox state via the reduced form glutathione/oxidized form glutathione (GSH/GSSG) ratio, influencing redox signaling pathways that regulate stress responses [90]. Therefore, managing S nutrition is arguably one of the most direct and powerful strategies for enhancing intrinsic Cd detoxification via the PC pathway and associated antioxidant defenses.

To systematically summarize the core mechanisms by which essential macronutrient elements alleviate Cd stress, the key processes, functional outcomes, and supporting references are compiled in Table 1.

## 4. Regulation of Cd Stress by Essential Micronutrient Elements and Non-Essential Beneficial Elements

### 4.1. Zinc

Zinc (Zn) exhibits a profound antagonistic relationship with Cd, primarily due to their chemical similarity (similar ionic radii and coordination chemistry) leading to shared uptake and transport pathways. Both ions compete for binding sites on ZRT, IRT-like protein (ZIP) family transporters (e.g., OsZIP1, OsZIP5, AtZIP1, AtZIP2, AtIRT1) and potentially other cation transporters like Nramps and HMAs [91,92]. Elevated Zn concentrations in the rhizosphere can significantly suppress Cd uptake by roots through this competitive inhibition [93]. Zn also influences the expression of metal transporter genes, as Zn deficiency often upregulates high-affinity ZIP transporters such as IRT1, which exhibit broad substrate specificity and may inadvertently enhance Cd uptake in the presence of Cd [94]. Conversely, adequate or elevated Zn supply can downregulate these transporters, reducing Cd influx [95]. Within the plant, Zn competes with Cd for binding to enzymes, transcription factors (e.g., Zn-finger proteins), and cellular ligands. Sufficient Zn protects essential metalloenzymes from Cd substitution and inactivation [96]. Zn is also a cofactor for key antioxidant enzymes, notably Copper (Cu)/Zn-SOD, and is involved in the synthesis of compounds like MTs, which can bind both Zn and Cd [97]. Zn application consistently enhances the activities of SOD, POD, CAT, and APX, and increases GSH and AsA levels in Cd-stressed plants, mitigating oxidative damage [98]. Furthermore, Zn influences Cd distribution, as adequate Zn can reduce Cd translocation from roots to shoots by enhancing root vacuolar sequestration or reducing xylem loading [99]. Molecular studies confirm that Zn application modulates the expression of genes involved in Cd uptake (*ZIPs*, *Nramps*), sequestration (*HMAs*, *MTPs*, *PCS*), and antioxidant defense [100,101]. Zn fertilization is therefore a cornerstone strategy for minimizing Cd accumulation in food crops like rice and wheat.

### 4.2. Iron

Fe homeostasis is intricately intertwined with Cd accumulation, particularly in strategy I plants (non-grass species), which rely on root reduction (ferric chelate reductase) and uptake of Fe^2+^ (IRT1) [102]. AtIRT1, the major high-affinity Fe^2+^ uptake transporter in *Arabidopsis*, also transports Cd^2+^, Mn^2+^, Zn^2+^, and other divalent cations with high efficiency [103]. Consequently, Fe deficiency dramatically upregulates *IRT1* expression, leading to a substantial increase in Cd uptake when Cd is present in the soil. This creates a critical vulnerability under low Fe availability. Conversely, adequate Fe supply suppresses *IRT1* expression, reducing Cd influx [104]. In strategy II plants (grasses), which utilize phytosiderophores (PS; mugineic acids) to chelate Fe^3+^ for uptake via yellow stripe-like (YSL) transporters, the relationship is more complex. While PS primarily chelate Fe^3+^, they can also bind other metals, including Cd, potentially facilitating its uptake under certain conditions [105]. However, Fe sufficiency generally reduces Cd accumulation in grasses as well [106]. Fe nutrition also impacts Cd-induced oxidative stress. Fe is a component of heme (catalase, peroxidases) and Fe-S cluster proteins involved in electron transport (photosynthesis, respiration). Cd can disrupt Fe homeostasis, inducing Fe-deficiency-like responses and potentially increasing free Fe pools that can catalyze Fenton reactions, amplifying ROS production [107]. Adequate Fe supply helps maintain proper functioning of Fe-containing antioxidant enzymes (CAT, various PODs) and minimizes Fe-catalyzed oxidative damage. Fe application has been shown to enhance antioxidant enzyme activities and reduce lipid peroxidation under Cd stress [108]. Furthermore, Fe plaque formation on rice roots, consisting of Fe (hydr)oxides precipitated by radial oxygen loss (ROL), can adsorb and immobilize Cd, reducing its uptake into the root [109]. Optimizing Fe nutrition, particularly in strategy I plants, is crucial for minimizing Cd uptake via the IRT1 pathway and mitigating secondary oxidative stress.

### 4.3. Manganese

Mn interacts with Cd through competitive uptake and its pivotal role in antioxidant defense, particularly within chloroplasts. Mn competes with Cd for uptake sites on root transporters, potentially including Nramps and ZIPs, leading to reduced Cd accumulation when Mn supply is sufficient. Mn deficiency can enhance Cd uptake and exacerbate its toxicity [110]. Crucially, Mn is an essential cofactor for Mn-SOD, the primary SOD isoform located in mitochondria and peroxisomes, and is also vital for the water-splitting complex (oxygen-evolving complex-OEC) of photosystem II (PSII) in chloroplasts [111]. Cd stress severely damages PSII, partly by displacing Mn^2+^/^3+^ from the OEC, disrupting oxygen evolution and leading to electron leakage and enhanced ROS generation. Adequate Mn supply protects the OEC structure and function, maintaining photosynthetic efficiency and reducing Cd-induced photoinhibition [112]. Enhanced Mn-SOD activity under sufficient Mn nutrition provides critical defense against superoxide radicals (O_2_^−^), particularly in organelles where Fe-SOD or Cu/Zn-SOD are absent or less prominent. Mn application consistently increases SOD and other antioxidant enzyme activities (CAT, POD) and reduces oxidative stress markers in Cd-exposed plants [113]. Mn also contributes to lignin biosynthesis via Mn-dependent peroxidases, potentially strengthening cell walls and creating additional binding sites for Cd, limiting its apoplastic movement and symplastic entry [114]. Studies indicate that Mn can reduce Cd translocation from roots to shoots [115]. Molecular evidence suggests Mn influences the expression of genes related to metal transport and antioxidant defense under Cd stress [116]. Therefore, maintaining adequate Mn nutrition is vital for protecting photosynthetic machinery, enhancing enzymatic antioxidant capacity, and reducing Cd uptake and oxidative damage.

### 4.4. Copper

The interaction between Cu and Cd involves competition for uptake and the role of Cu as a cofactor in key antioxidant enzymes and ET signaling. Cu and Cd may compete for binding sites on transporters like copper transporters (COPTs), ZIPs, and potentially HMAs [117]. Elevated Cu levels can sometimes reduce Cd uptake, although this interaction is often less pronounced than with Zn or Fe [118]. More significantly, Cu is a critical component of antioxidant defenses. It is the cofactor for Cu/Zn-SOD (cytosolic and chloroplastic isoforms), crucial for dismutating O_2_^−^ to H_2_O_2_ and O_2_ [119]. Cu is also a cofactor for various oxidases (e.g., ascorbate oxidase, polyphenol oxidases) and is involved in the electron transport chain (plastocyanin). Cd stress can disrupt Cu homeostasis, potentially impairing the function of Cu/Zn-SOD and other cuproenzymes, thereby compromising antioxidant capacity [120]. Adequate Cu supply helps maintain Cu/Zn-SOD activity, reducing oxidative stress under Cd exposure [121]. Recent studies have shown that Cu regulates ET signaling by interacting with ET receptors [122]. ET is a key signaling molecule in plant responses to toxic metal stress, including Cd, mediating processes like root growth inhibition, aerenchyma formation, and defense gene activation [123]. Perturbation of Cu status could therefore influence ET signaling pathways involved in Cd tolerance. While essential in optimal amounts, excess Cu itself is toxic. Careful management of Cu nutrition is necessary to support antioxidant defenses and signaling without inducing Cu toxicity.

### 4.5. Silicon

Silicon (Si), while not considered essential for most higher plants, is highly beneficial and demonstrates remarkable efficacy in alleviating Cd toxicity across diverse species, though mechanistic evidence for direct genetic, biochemical, or physiological effects of monomeric silicic acid remains limited. Si accumulates predominantly in the apoplast, forming silica phytoliths and Si-cell wall complexes—consistent with the apoplastic obstruction hypothesis proposed by Coskun et al. [124], which attributes Si’s benefits to physical barriers rather than direct biochemical interactions [125]. Its primary mechanisms for Cd mitigation involve: (1) Reduced Cd bioavailability: Si application can increase soil pH (especially with silicate materials), promoting Cd adsorption onto soil colloids and precipitation [126]. In the rhizosphere, Si can compete with Cd for adsorption sites on root surfaces. (2) Enhanced physical barriers: Si deposition in cell walls (particularly endodermis, epidermis, vascular bundles) increases their mechanical strength and creates additional negatively charged sites (silicic acid polymers) that bind Cd^2+^, impeding apoplastic Cd transport and reducing symplastic uptake [127]. (3) Complexation/Compartmentalization: Si can stimulate the synthesis of lignin and callose, thickening cell walls and casparian strips, further restricting Cd movement [128]. Si also promotes the compartmentalization of Cd within root and leaf cells, potentially by enhancing vacuolar sequestration or forming less toxic Si-Cd complexes or co-precipitates within cell walls or vacuoles [129]. (4) Improved antioxidant defense: Si application consistently enhances the activities of key antioxidant enzymes (SOD, POD, CAT, APX, GR) and elevates levels of non-enzymatic antioxidants (AsA, GSH, phenolics), significantly reducing Cd-induced oxidative stress markers like H_2_O_2_ and MDA [130]. (5) Regulation of transporter genes: Si downregulates the expression of genes encoding Cd influx transporters and upregulates genes involved in Cd efflux and antioxidant defense [131]. Si also modulates phytohormone levels (e.g., increasing JA, SA; decreasing ET) involved in Cd stress signaling [132]. The multifaceted nature of Si-mediated alleviation makes it a highly promising amendment for Cd-contaminated agriculture.

### 4.6. Selenium

Selenium (Se), a non-essential but highly beneficial element for most higher plants, exerts complex, dose-dependent effects on Cd stress, primarily through modulation of antioxidant systems, formation of Cd-Se complexes, and influence on sulfur metabolism [133]. At low, non-toxic doses (typically 1–10 µM), Se acts as a potent antioxidant. It can directly scavenge ROS, enhance the activity of antioxidant enzymes (glutathione peroxidase-GPX, which can utilize thioredoxin or GSH; also SOD, CAT, APX), and regenerate other antioxidants like AsA. Se is incorporated into selenoproteins, including various isoforms of GPX, which play crucial roles in reducing H_2_O_2_ and lipid hydroperoxides [134]. Se application significantly increases GSH levels and the GSH/GSSG ratio, bolstering the AsA-GSH cycle [135]. Furthermore, Se can form inert or less toxic complexes with Cd, such as Cd-Se nanocrystals, potentially reducing free Cd^2+^ activity in cells and facilitating vacuolar sequestration [136]. As a chemical analog of S, Se interacts with S metabolism by being assimilated through the S pathway and forming Selenocysteine (SeCys) and Selenomethionine (SeMet). This can influence the availability of S for GSH and PC synthesis [137]. While moderate Se often enhances PC production under Cd stress, high Se can compete with S for uptake and assimilation, potentially limiting GSH synthesis [138]. Se influences the expression of genes involved in Cd uptake (*Nramps*, *IRTs*), transport (*HMAs*), chelation (*PCS*), and antioxidant defense (*GPX*, *SOD*, *CAT*, *GR*) [139]. However, although the effects of Se are highly concentration-dependent, supra-optimal levels become toxic. Careful optimization of Se application is essential for exploiting its protective benefits against Cd toxicity without causing selenosis.

Similarly, the multifaceted roles of micronutrients in mitigating Cd toxicity—including competitive uptake, antioxidant activation, and transporter regulation—along with their supporting references, are synthesized in Table 2.

## 5. Molecular Mechanisms of Nutrient-Mediated Cd Tolerance

The intricate interplay between nutrient elements and Cd stress is orchestrated at the molecular level through complex transcriptional and post-transcriptional regulatory networks, signal transduction cascades, and epigenetic modifications.

### 5.1. Transcriptional Regulation

Nutrient availability profoundly influences the expression of genes central to Cd response through the regulation of key transcription factor (TF) families. For instance, bZIP TFs, are regulated by cellular redox status and S availability via GSH. These TFs control S deficiency responses and regulate the expression of both SULTR transporters and genes involved in GSH biosynthesis (*GSH1*, *GSH2*), thereby directly linking sulfur status to Cd chelation capacity [140,141,142]. MYB TFs participate in diverse stress responses and secondary metabolism, and some members regulate metal transporter genes such as *Nramps* and *HMAs*, as well as *PCS* [143]. Their expression can also be modulated by nutrient status, including P or Fe deficiency. WRKY TFs, major regulators of plant defense responses to toxic metal stress, are activated by ROS, Ca^2+^ signals, and MAPK cascades induced by Cd. They in turn regulate genes related to antioxidant enzymes, pathogenesis-related proteins, and metal transporters [144], with their expression or activity further influenced by nutrient signals. NAC TFs, which function in senescence and stress responses, regulate genes associated with cell wall modification, antioxidant defense, and metal transport under Cd stress [145], and they are also affected by N availability. Additionally, zinc-finger proteins (ZIP TFs) are directly regulated by Zn status. Zn deficiency induces Zn-deficiency responsive TFs, including bZIP19/23 and F-group bZIPs, that upregulate high-affinity Zn uptake transporters (ZIP family), potentially increasing Cd uptake due to the shared transport pathways [146].

Nutrient deficiencies or sufficiencies often act as signals that modulate the activity of these TFs. For instance, Fe deficiency strongly induces FER-like iron-deficiency-induced transcription factor (FIT), which upregulates *IRT1* expression, inadvertently increasing Cd uptake [147]. S deficiency induces SULFUR LIMITATION 1 (SLIM1), an EIL-type TF regulating S assimilation and GSH synthesis genes [148]. Adequate S supply represses SLIM1, optimizing GSH production for Cd chelation [149]. Ca^2+^ signals, decoded by CaM/CMLs, CDPKs, and CBL-CIPKs, directly phosphorylate and activate TFs regulating stress-responsive genes [150]. The CBL-CIPK pathway, particularly CIPK23, regulates transporters including IRT1 (phosphorylation affecting stability/activity) and potentially others involved in Cd handling [151].

### 5.2. Signaling Pathways

Nutrient status is integrated with Cd stress perception through several key signaling hubs. Ca signaling serves as a primary mechanism, wherein Cd-induced cytosolic Ca^2+^ spikes are decoded by sensor proteins such as CaMs, CBLs, and CDPKs, leading to the activation of downstream responses including gene expression and antioxidant defense [152]. The activity of Ca^2+^ channels can be influenced by nutrient cations such as K^+^ and Mg^2+^ through modulation of membrane potential, while S status, via GSH-mediated redox balance, may affect the sensitivity of Ca^2+^ signaling [153]. ROS signaling also plays a critical role. Cd-induced ROS act as signaling molecules that activate MAPK cascades and redox-sensitive TFs [154]. Nutrient-enhanced antioxidant systems, including GSH (dependent on S), SOD (dependent on Cu/Zn or Mn), CAT (dependent on Fe), and GPX (dependent on Se), modulate the amplitude and duration of ROS signals, thereby maintaining them at non-toxic levels without compromising signaling functions [155]. Hormone signaling is another integrative node. Cd stress perturbs the balance of JA, SA, ET, ABA, and auxins [156]. Nutrient availability influences hormone biosynthesis and signaling. For instance, Cu regulates ET signaling by interacting with ET receptors, N and S affect JA and SA synthesis, and Ca participates in ABA signaling [157], ultimately regulating TFs and defense-related gene expression. Furthermore, conserved nutrient-sensing hubs such as the target of rapamycin (TOR) kinase and SNF1-Related Kinase 1 (SnRK1) integrate information on nutrient and energy status, particularly carbon, N, and S, with stress signaling [158]. Under favorable nutrient conditions, TOR promotes growth, whereas SnRK1 activates stress responses and catabolism during energy deficiency. Cd stress likely modulates these kinases, with nutrient availability shaping the trade-off between growth and defense [159]. Recent evidence indicates SnRK1 can phosphorylate TFs involved in metal homeostasis and antioxidant defense. Finally, microRNAs (miRNAs) contribute to post-transcriptional regulation under Cd stress by targeting transcripts encoding metal transporters, TFs, and enzymes related to antioxidant synthesis and hormone signaling [160]. The expression of specific miRNAs is influenced by nutrient availability, such as S, N, or P, adding another layer of nutrient-dependent control. For example, miR395, which is up-regulated under S deficiency, targets ATP sulfurylase genes and indirectly affects GSH biosynthesis, thereby linking S nutrition to Cd detoxification capacity [161,162].

### 5.3. Epigenetic Regulation

Emerging evidence points to epigenetic mechanisms (DNA methylation, histone modifications, chromatin remodeling) in mediating long-term adaptive responses to Cd stress, potentially influenced by nutrient status [163]. Cd exposure can alter global and gene-specific DNA methylation patterns and histone marks (e.g., H3K9ac, H3K4me3, H3K27me3) associated with stress-responsive genes [164]. Nutrients involved in one-carbon metabolism (e.g., SAM, derived from methionine/S) are the methyl donors for DNA and histone methylation [165]. Therefore, S and N nutrition, by affecting SAM availability, could potentially influence the epigenetic landscape and the heritable expression of Cd tolerance genes. Understanding these complex epigenetic–nutrient–Cd interactions is a frontier research area.

## 6. Nutrient Interactions and Synergistic Effects

The influence of nutrients on Cd stress is rarely isolated, as complex interactions, such as synergism, antagonism, and additivity, occur among nutrients themselves and significantly shape the overall plant response. Understanding these interactions is essential for designing effective nutrient management strategies. For instance, S and N exhibit strong synergy in Cd detoxification through the PC pathway. Adequate N supply provides glutamate as the carbon skeleton for GSH synthesis, while S supplies the cysteine moiety. Co-application of S and N fertilizers often leads to greater increases in GSH, PC levels, Cd chelation, and overall tolerance compared to single nutrient applications, supported by coordinated upregulation of genes involved in N and S assimilation and GSH synthesis [166]. Ca and P also interact meaningfully. In the soil, they can co-precipitate Cd as insoluble compounds, while within the plant, Ca contributes to membrane stability and defense signaling, and P supports energy-dependent detoxification processes. Combined application of Ca and P can in some contexts more effectively reduce Cd uptake and toxicity than either nutrient alone [167]. Among micronutrients, Zn, Fe, and Mn often compete during uptake, Zn or Fe deficiency upregulates metal transporters such as ZIP and IRT1, thereby increasing Cd accumulation. Conversely, excessive Zn may induce Fe or Mn deficiency, undermining Cd mitigation benefits and causing secondary stress. Therefore, balanced ratios of these micronutrients are critical [168]. Si and Se can act complementarily, with Si mainly enhancing physical barriers and reducing Cd translocation, and Se improving antioxidant capacity and intracellular Cd complexation. Their co-application has shown additive or synergistic effects in mitigating Cd toxicity [169]. K and Mg work synergistically to support osmotic regulation, enzyme function, and photosynthesis, and an appropriate K:Mg ratio is vital for maintaining plant vigor under Cd stress [170]. Furthermore, nutrient amendments influence rhizosphere microbial community structure and function. Certain microbes improve the availability of nutrients such as P, Fe, and Zn, while others immobilize Cd through sorption, pH modulation, or siderophore production [171,172]. This interplay highlights the importance of considering nutrient-microbiome interactions in Cd management strategies. Given these multifaceted interactions, a holistic fertilization approach, that accounts for native soil fertility, plant nutrient demands, potential antagonisms, and specific remediation objectives such as exclusion, stabilization, or extraction, is necessary for effective Cd stress mitigation.

## 7. Applied Perspectives: Nutrient Management for Cd Mitigation

Harnessing nutrient regulation offers practical, cost-effective, and environmentally friendly strategies for managing Cd contamination in agriculture and enhancing environmental remediation.

### 7.1. Fertilization Strategies

Tailored fertilizer formulations can be designed to achieve specific rhizosphere effects that mitigate Cd availability. For example, selecting nitrate-based over ammonium-based N sources can alkalinize the rhizosphere and reduce Cd solubility [173], while the application of P fertilizers promotes Cd precipitation in suitable soils. Silicate fertilizers, such as calcium silicate slag, or Si-rich amendments including rice husk biochar, can effectively supply Si for enhanced Cd resistance [174]. Optimizing the timing and placement of fertilizers further improves their efficacy. Split application of N, deep placement of P, and band application of Zn or Si can enhance nutrient use efficiency and locally reduce Cd bioavailability around the root zone [175]. Maintaining balanced nutrition is essential to avoiding deficiencies that heighten susceptibility to Cd uptake, such as Fe in strategy I plants or Zn, while also preventing nutrient toxicities or imbalances, like Zn deficiency induced by excess P. Regular soil and plant tissue analyses provide critical guidance for sustaining adequate levels of Ca, Mg, K, and S, which collectively support plant health and Cd detoxification mechanisms. Finally, combining amendments often yields synergistic effects, such as S with N to boost PC synthesis, Si with Se for joint physical and antioxidant benefits, or organic amendments like compost and manure that supply multiple nutrients and improve soil cation exchange capacity and organic matter content for Cd immobilization [176,177].

### 7.2. Breeding and Biotechnology

Developing nutrient-efficient cultivars represents a promising strategy for reducing Cd accumulation. This involves breeding genotypes with enhanced ability to acquire and utilize mitigating nutrients, such as S for PC synthesis, Zn to compete with Cd uptake, or Si through favorable root traits, under Cd stress conditions [178]. Beyond conventional breeding, genetic engineering offers tools to manipulate transporter expression, such as knocking down broad-specificity Cd influx transporters (e.g., OsNramp5 in rice) or enhancing the expression of vacuolar sequestration transporters (e.g., OsHMA3) and efflux transporters. However, modifying transporter expression carries risks: for example, knocking down OsNramp5 (a major Cd uptake transporter) concurrently reduces Mn absorption, leading to Mn deficiency and impaired photosynthesis [179]. Precise, tissue-specific modulation of transporters is therefore required to avoid unintended nutritional imbalances. Modifying nutrient transporter expression can further improve competitive exclusion of Cd, for instance by elevating high-affinity Zn uptake systems [180]. Another approach involves enhancing chelation capacity through overexpression of genes involved in GSH synthesis (*GSH1*, *GSH2*) or PCS, particularly when combined with sufficient S and N supply [181]. Improving antioxidant defense is also essential. This can be achieved by engineering plants to overexpress antioxidant enzymes (e.g., SOD, APX, GPX) or boosting non-enzymatic antioxidants like ascorbate and GSH, supported by optimal supply of essential metal cofactors such as Cu, Zn, Mn, Fe, and Se. Finally, targeted gene editing using CRISPR/Cas9 and related technologies enables precise modification of genes governing Cd uptake, translocation, sequestration, and nutrient-Cd interactions, offering unprecedented potential for developing crops with tailored Cd resistance [182].

### 7.3. Phytoremediation Enhancement

In phytoremediation approaches, tailored nutrient management is essential for optimizing efficiency. For hyperaccumulator species such as *Sedum alfredii*, *Noccaea caerulescens*, and *Arabidopsis halleri*, supplied with optimal levels of S, N, Ca, and K, plant growth, biomass production, and Cd accumulation capacity can be significantly enhanced, thereby improving phytoextraction performance [183]. Careful management of micronutrients is also necessary to prevent deficiencies that could compromise hyperaccumulation. Alternatively, nutrient-assisted phytoextraction offers a more environmentally sustainable strategy than synthetic chelants such as EDTA or EDDS, which pose ecological risks. Amendments such as ammonium sulfate, which acidifies the soil and supplies N and S, or Si- and Se-based formulations can promote Cd mobilization and translocation in selected plants without introducing persistent contaminants [184]. In contrast, phytostabilization aims to reduce Cd mobility and bioavailability in soil through combined nutrient and amendment strategies. These include liming with calcium carbonate to increase pH, applying P to precipitate Cd, and incorporating organic amendments to enhance complexation and adsorption. Notably, Cd encapsulation in phytoliths (amorphous silica precipitates in plants) can facilitate long-term Cd sequestration in soil reducing Cd bioavailability and mobility in soil [185]. When implemented along with suitable plant species possessing dense root systems, such as certain grasses or willows, these measures help immobilize Cd, minimize leaching, and mitigate ecological transfer [186].

### 7.4. Limitations of Cd Mitigation Strategies

Despite their practical potential, nutrient-based Cd mitigation strategies face inherent limitations. Optimized fertilization is strongly soil dependent: for example, P-induced Cd precipitation is ineffective in acidic soils (pH < 5.5) due to low phosphate solubility [187], and NH_4_^+^-based N fertilizers may exacerbate Cd uptake by acidifying rhizospheres [35]. Breeding and biotechnology approaches carry trade-offs: enhancing Zn uptake to compete with Cd may reduce absorption of other micronutrients like Fe [188], and genetically modified crops face regulatory and public acceptance barriers in many regions. Phytoremediation is time intensive (requiring 3–10 growing seasons for meaningful Cd removal) and constrained by climate (e.g., low biomass in arid regions) [189]. Additionally, excessive nutrient amendments can cause secondary issues: surplus Si may inhibit P uptake [190], and high Se doses induce selenosis in plants and herbivores [191].

## 8. Conclusions and Future Perspectives

The intricate orchestration of plant responses to Cd stress is profoundly modulated by the status and availability of essential nutrient elements. This review has elucidated the diverse and sophisticated mechanisms through which essential macronutrient elements (N, P, K, Ca, Mg, S), essential micronutrient elements (Zn, Fe, Mn, Cu) and non-essential beneficial elements (Si, Se) influence Cd behavior from the rhizosphere to the subcellular level. Nutrients impact Cd bioavailability, compete for uptake and binding sites, form complexes, stabilize cellular structures, activate and enhance antioxidant defense systems, regulate signal transduction cascades, and control the expression of genes critical for Cd transport, chelation, sequestration, and detoxification. The interactions between nutrients themselves add another layer of complexity, necessitating a holistic approach to nutrient management. The practical implications are significant: optimized fertilization strategies, informed by a deep understanding of these nutrient–Cd interactions, offer powerful tools for reducing Cd accumulation in food crops, thereby enhancing food safety, and for improving the efficiency of phytoremediation technologies aimed at cleaning up Cd-contaminated environments.

Future research should prioritize several emerging frontiers to advance the understanding and management of Cd stress in plants. A key direction is elucidating the spatiotemporal dynamics of nutrient and Cd fluxes across the root-soil interface and within plant tissues using advanced imaging technologies such as synchrotron-based X-ray fluorescence microscopy (XFM) and nanoscale secondary ion mass spectrometry (NanoSIMS). Simultaneously, it is essential to decipher the molecular crosstalk between nutrient-signaling pathways, including TOR, SnRK1, and nutrient-specific TF networks, and Cd stress responses, to understand how plants reallocate resources and prioritize defense mechanisms. Further investigation is needed into how nutrient status influences epigenetic modifications such as DNA methylation and histone marks, as well as miRNA expression, which collectively regulate long-term Cd tolerance and potential transgenerational effects. The interplay between nutrient amendments and the rhizosphere microbiome also demands deeper exploration, particularly how specific microbial consortia respond to nutrient inputs and subsequently affect Cd bioavailability and speciation. From an applied perspective, efforts should focus on developing precision nutrient management strategies that use sensor technologies and data analytics to tailor interventions to specific soil properties, Cd levels, and crop varieties, whether the goal is to minimize Cd in grains or enhance phytoextraction. Concurrently, advanced biotechnological approaches can be leveraged to design smart plants with optimized traits, such as reduced Cd uptake through transporter manipulation, improved vacuolar sequestration, increased nutrient use efficiency for elements like S, Si, Zn, and Ca, and enhanced antioxidant capacity. Finally, the development of novel synergistic soil amendments that combine nutrient supply with Cd immobilization capabilities, such as engineered biochars, clay minerals, and iron oxides, will support sustainable and effective Cd mitigation.

The profound link between plant nutrition and Cd stress resilience underscores that managing nutrient elements is not merely about maximizing yield, but is fundamentally intertwined with managing environmental contaminants and ensuring the safety and sustainability of our agricultural systems. A nutrient-centric approach, grounded in a deep mechanistic understanding, holds immense promise for addressing the pervasive challenge of Cd pollution.

## Figures and Tables

**Table 1:** Key mechanisms of essential macronutrient elements in alleviating Cd stress in plants.

**Elements**	**Key Mechanisms of Cd Stress Alleviation**	**References**
N	1. NO_3_^−^-mediated rhizosphere alkalinization reduces Cd solubility; NH_4_^+^ causes acidification (opposite effect). 2. Enhances GSH/PC synthesis via GS/GOGAT cycle for Cd chelation. 3. Maintains photosynthetic efficiency and antioxidant enzyme (SOD, APX) activity.	[33,34,42]
P	1. Precipitates Cd as insoluble Cd_5_(PO_4_)_3_OH/Cd_3_(PO_4_)_2_ in soil, reducing bioavailability. 2. Supports ATP-dependent vacuolar sequestration (via HMAs/ABCCs). 3. Enhances antioxidant defense (SOD, POD) and membrane stability.	[45,46,51,53]
K	1. Competes with Cd^2+^ for cell wall/membrane binding sites (similar hydrated radius). 2. Maintains membrane integrity via H^+^-ATPase, reducing K^+^ leakage and Cd influx. 3. Activates ROS-scavenging enzymes (e.g., SOD) and modulates Cd transporter (HMA) expression.	[55,59,60,63]
Ca	1. Competes with Cd^2+^ for root transporters (e.g., annexins) and cell wall pectin binding sites. 2. Stabilizes plasma membrane to inhibit Cd-induced lipid peroxidation. 3. Triggers Ca^2+^ signaling (CBL-CIPK pathway) to activate Cd detoxification genes (*PCS*, *HMA*).	[64,65,66,70]
Mg	1. Protects chloroplast structure and photosynthetic apparatus from Cd damage. 2. Acts as cofactor for GSH synthesis (γ-ECS, GS) and AsA-GSH cycle enzymes (GR). 3. Reduces Cd accumulation in photosynthetic tissues via competitive uptake.	[75,76,80,81]
S	1. Precursor for cysteine, GSH, and PCs (key Cd chelators) via ATPS/APR-mediated assimilation. 2. Enhances vacuolar sequestration of Cd-PC complexes (via ABCC transporters). 3. Modulates redox homeostasis via GSH/GSSG ratio.	[82,84,86,87]

**Table 2:** Key mechanisms of essential micronutrient elements and non-essential beneficial elements in alleviating Cd stress in plants.

**Elements**	**Key Mechanisms of Cd Stress Alleviation**	**References**
Zn	1. Competes with Cd^2+^ for ZIP/IRT/Nramp transporters (e.g., OsZIP1, AtIRT1) to reduce Cd uptake. 2. Downregulates Cd influx transporters (ZIPs) under sufficient supply. 3. Enhances Cu/Zn-SOD activity and GSH levels to mitigate oxidative stress.	[91,93,95,98]
Fe	1. Suppresses *IRT1* expression (major Cd/Fe transporter) in strategy I plants, reducing Cd uptake. 2. Maintains Fe-containing antioxidant enzymes (CAT, POD) to inhibit Fenton reaction-induced ROS. 3. Fe plaque on rice roots adsorbs and immobilizes Cd.	[102,103,104,109]
Mn	1. Protects photosystem II (OEC) by preventing Cd-induced Mn displacement, reducing photoinhibition. 2. Activates Mn-SOD (mitochondrial/peroxisomal) to scavenge O_2_^−^. 3. Enhances lignin biosynthesis (via Mn-peroxidases) to restrict apoplastic Cd movement.	[111,112,113,116]
Cu	1. Cofactor for Cu/Zn-SOD (cytosolic/chloroplastic) to dismutate O_2_^−^. 2. Regulates ET signaling by interacting with receptors. 3. Competes with Cd for COPT/ZIP transporters (minor antagonistic effect).	[117,119,120,122]
Si	1. Increases soil pH and Cd adsorption onto colloids; deposits in cell walls to bind Cd^2+^. 2. Downregulates Cd influx transporters (OsNramp5, OsIRT1) and upregulates vacuolar sequestration (OsHMA3). 3. Enhances antioxidant enzyme (SOD, CAT) activity and modulates phytohormones (JA/SA).	[125,126,127,131]
Se	1. Scavenges ROS directly and activates GPX/SOD to reduce lipid peroxidation. 2. Forms inert Cd-Se complexes and enhances vacuolar sequestration. 3. Modulates S metabolism (SeCys/SeMet) to influence GSH/PC synthesis (dose-dependent).	[133,134,135,139]

## Data Availability

Not applicable.
